# Structural, Magnetic, and Electrical Properties of CoFe_2_O_4_ Nanostructures Synthesized Using Microwave-Assisted Hydrothermal Method

**DOI:** 10.3390/ma15227955

**Published:** 2022-11-10

**Authors:** Shalendra Kumar, Faheem Ahmed, Nagih M. Shaalan, Rajesh Kumar, Adil Alshoaibi, Nishat Arshi, Saurabh Dalela, Fatima Sayeed, Sourabh Dwivedi, Kavita Kumari

**Affiliations:** 1 Department of Physics, College of Science, King Faisal University, P.O. Box 400, Al-Ahsa 31982, Saudi Arabia; 2 Department of Physics, University of Petroleum & Energy Studies, Dehradun 248007, Uttarakhand, India; 3 Physics Department, Faculty of Science, Assiut University, Assiut 71516, Egypt; 4 University School of Basic and Applied Sciences, Guru Gobind Singh Indraprastha University, New Delhi 110078, India; 5 Department of Basic Sciences, Preparatory Year Deanship, King Faisal University, P.O. Box 400, Al-Ahsa 31982, Saudi Arabia; 6 Department of Pure & Applied Physics, University of Kota, Kota 324005, Rajasthan, India; 7 Basic Science Department, Pre-Professional Program-Female, College of Science and Health Profession, King Saud bin Abdul Aziz University for Health Sciences, Al-Ahsa 31982, Saudi Arabia; 8 Department of Applied Physics, Aligarh Muslim University, Aligarh 202001, Uttar Pradesh, India; 9 School of Materials Science and Engineering, Changwon National University, Changwon 51140, Gyeongnam, Korea

**Keywords:** CoFe_2_O_4_, X-ray diffraction, DC magnetization, FE-SEM, dielectric spectroscopy

## Abstract

Magnetic nanostructures of CoFe_2_O_4_ were synthesized via a microwave-assisted hydrothermal route. The prepared nanostructures were investigated using X-ray diffraction (XRD), field emission electron microscopy (FE-SEM), energy dispersive X-ray (EDX) spectroscopy, high-resolution transmission electron microscopy (HR-TEM), selective area electron diffraction (SAED) pattern, DC magnetization, and dielectric spectroscopy measurements. The crystal structure studied using HR-TEM, SAED, and XRD patterns revealed that the synthesized nanostructures had a single-phase nature and ruled out the possibility of any secondary phase. The lattice parameters and unit cell volume determined from the XRD data were found to be 8.4821 Å and 583.88 Å^3^. The average crystallite size (~7.0 nm) was determined using Scherrer’s equation. The FE-SEM and TEM micrographs revealed that the prepared nanostructures had a spherical shape morphology. The EDX results showed that the major elements present in the samples were Co, Fe, and O. The magnetization (M) versus temperature (T) measurements specified that the CoFe_2_O_4_ nanostructures showed ferromagnetic ordering at room temperature. The blocking temperature (T_B_) determined using the M-T curve was found to be 315 K. The magnetic hysteresis (M-H) loop of the CoFe_2_O_4_ nanostructures recorded at different temperatures showed the ferromagnetic behavior of the CoFe_2_O_4_ nanostructures at temperatures of 200 K and 300 K, and a superparamagnetic behavior at 350 K. The dielectric spectroscopy studies revealed a dielectric constant (ε′) and loss tangent (tanδ) decrease with the increase in the frequency, as well as demonstrating a normal dispersion behavior, which is due to the Maxwell–Wagner type of interfacial polarization. The values of ε′ and tanδ were observed to increase with the increase in the temperature.

## 1. Introduction

Magnetic nanostructures have received widespread attention from researchers, due to their underlying chemical and physical properties, as well as for potential technological applications [1,2,3,4,5,6,7]. These magnetic nanostructures display remarkable electrical and magnetic properties compared to their bulk counterparts. It has been observed that magnetic nanostructures have numerous exceptional features in the nanometer range, due to strong modifications occurring at in surface/interfaces as compared to the bulk material. Magnetic materials display various unique properties adaptable to modulations, such as saturation magnetization, superparamagnetism, coercivity, spin-glass behavior, etc. [8,9,10,11,12]. Moreover, the design and growth of magnetic nanostructures have become a central point for researchers in applied research, because of their unusual and superior structural properties. In particular, spinel ferrite with an AB_2_O_4_ type crystal structure has shown remarkable magnetic and dielectric properties in its nanocrystalline shape compared to bulk particles and has demonstrated technological applications in various fields, such as for biomedical fields, chemical stability, storage media, high electrical resistivity, magnetic nanofluids, high-pressure sensors through Cr^3+^ doping, magnetic resonance imaging, mechanical hardness, multilayer chip indicators, super-hard materials, high-temperature ceramics, etc. [13,14,15,16,17,18,19]. Furthermore, it has been observed that, owing to their excellent dielectric properties, spinel ferrites possess numerous applications in radio, as well as microwave, frequency devices. Notably, the dielectric and magnetic properties of these ferrites show sensitivity towards the method of preparation and sintering conditions. Many groups have explored ferrites in the nanodomain widely because they demonstrate enhanced properties at low grain sizes [20,21,22,23]. Various techniques, such as sol-gel, hydrothermal, solvothermal, and microwave hydrothermal, etc., have developed for spinel ferrite nanostructures [24,25,26]. CoFe_2_O_4_ is a spinel ferrites that is an eminent hard magnetic material with ferromagnetic behavior, with a high Curie temperature (~793 K), strong magnetocrystalline anisotropy along with a moderate magnetization (*M_s_*), and high coercivity (*H_c_*). These properties of CoFe_2_O_4_ indicate its suitability for magnetic recording applications, in high-density digital recording disks, etc. The ferromagnetic ordering in CoFe_2_O_4_ results from the super-exchange interaction between the cations located in octahedral/tetrahedral sites and the oxygen anion. In this study, CoFe_2_O_4_ nanostructures were synthesized using a microwave-assisted hydrothermal procedure. The prepared nanostructures were studied using X-ray diffraction, dielectric spectroscopy, and the dc magnetization method. 

## 2. Experimental

The microwave-assisted hydrothermal technique was employed in the synthesis of CoFe_2_O_4_ nanostructures. All chemicals were of analytical grade, purchased from Sigma–Aldrich, and used without any further treatment. In a typical synthesis, a solution of 2:1 stoichiometric molar ratio of Iron (III) nitrate nonahydrate and Cobalt(II) nitrate hexahydrate was dissolved in 50 mL de-ionized water under constant magnetic starring. Thereafter, the solution was made alkaline by adding NH_4_OH dropwise to reach a pH level of 9. After stirring for 1 h at room temperature, the solution was finally transferred into a Teflon vessel and was kept at 200 °C in a microwave hydrothermal for 5 min. The solution was allowed to cool at room temperature after microwave processing. The precipitate obtained via centrifugation was washed using deionized water and absolute ethanol five times and separated by centrifugation at 4000 rpm. Finally, the precipitate was dried in a hot oven at 80 °C for 2 h. The phase of the as-obtained product was characterized by X-ray diffraction (XRD) using a Phillips X’pert (MPD-3040) diffractometer (λ_Cu_*_Kα_* = 1.5406 Å) operated at a voltage of 40 kV and current of 30 mA. Field emission scanning electron microscopy (FESEM) images were obtained using a MIRA II LMH microscope. The contribution of elements in CoFe_2_O_4_ was determined by energy-dispersive X-ray spectroscopy (EDX, Inca Oxford, attached to the FESEM). The magnetic properties were studied using quantum designed PPMS. The dielectric spectroscopy studies were carried out using an Alpha-A High-Performance Frequency Analyzer. The dielectric measurements were performed at different frequencies, ranging from 1.0 Hz to 10 MHz. Before the dielectric measurements, a circular pallet of material was prepared with a diameter and thickness of ~1.0 cm and 1.5 mm, respectively. The electrode was prepared using silver paint, applied on both parallel sides of the pallet.

## 3. Results and Discussion

**Figure 1:**
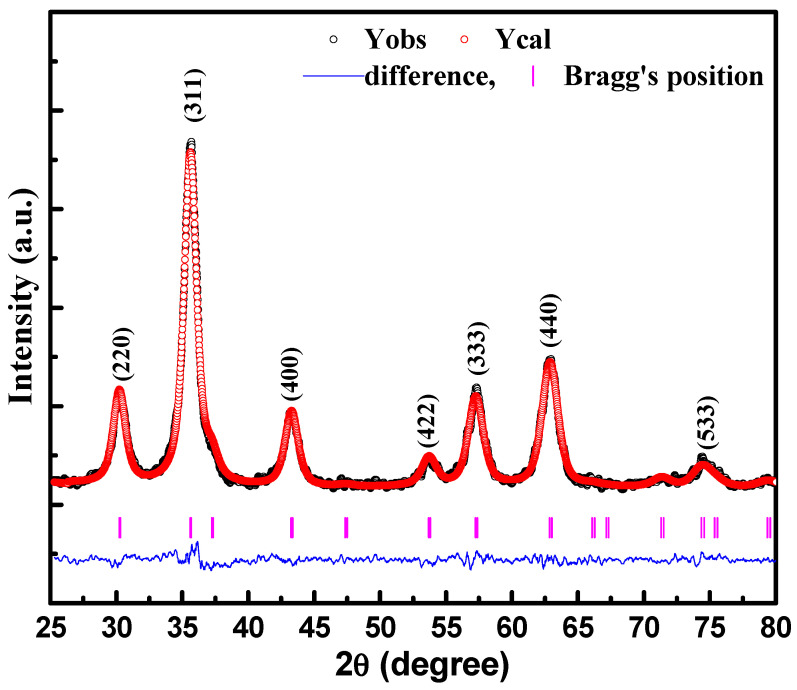
X-ray diffraction pattern of CeFe_2_O_4_ nanostructures measured at room temperature.

The crystalline structure and phase of the CoFe_2_O_4_ nanostructures were characterized using room temperature XRD measurements, as shown in Figure 1. The Fullproof program unitized for the Rietveld refinement process clearly inferred that the CoFe_2_O_4_ nanostructures had a single-phase nature with phase face-centered cubic (FCC) geometry and excluded the existence of any secondary phase. The theoretical and experimental data points are symbolized by red-colored and black-colored circles, respectively. Bragg’s positions corresponding to the FCC structure are highlighted using vertical lines of link color. The difference between experimental and theoretical data is represented by the blue lines. The value of χ^2^, which describes the quality and reliability of refinement, was 1.2. It is worth mentioning here that all the XRD peaks showed broadening, which was attributed to the nanocrystalline nature of the CoFe_2_O_4_ nanostructures. The diffraction peaks observed at 2θ = 29.92°, 35.34°, 42.9°, 57.43°, and 62.44°, and corresponding to (220), (311), (400), (333), and (440) planes were assigned to the spinel structure of CoFe_2_O_4_ nanostructures with the space group Fd3m. All the diffraction peaks indexed in the XRD pattern were analogous to the JCPDS card number 22–1086 [27]. The average particle size, D, of CeF_2_O_4_ nanostructures was determined using the highest intense peak (311), using Scherrer’s equation, D=0.9λβ Cosθ [28], where *β* is the broadening of the diffraction peak at angle *θ*. The *β* was measured using the equation β=(β02−B2)12, where $\beta_{0}$ highlights the full width at half maximum (FWHM) of the diffraction peaks and *B* refers to the instrumental broadening. The λ is the wavelength (1.5406 Å) of the X-ray used to record the pattern. The diffraction peaks were fit by the mean of the Voigt function. The particle size evaluated using the high-intensity low angle (311) and high angle (440) peaks were found to be ~7.0 nm. The lattice parameter (a) was also calculated using the formula a=dhkl(h2+k2+l2), here (*h*, *k*, *l*), represents the miller indices whereas *d_hkl_* denotes the interplaner spacing determined using the XRD pattern. The lattice parameter estimated using the (311) peak was found to be 8.3581 Å. Furthermore, the X-ray density (theoretical) was also calculated using the formula in [29], *d_hkl_* = 8*M*/*Na*^3^, where *M* stands for the molecular weight of CoFe_2_O_4_ and N denotes the Avogadro number, whereas *a* highlights the lattice constant and where 8 represents the number of atoms per unit cell. The X-ray density was found to be ~5.649 g/cm^3^.

**Figure 2:**
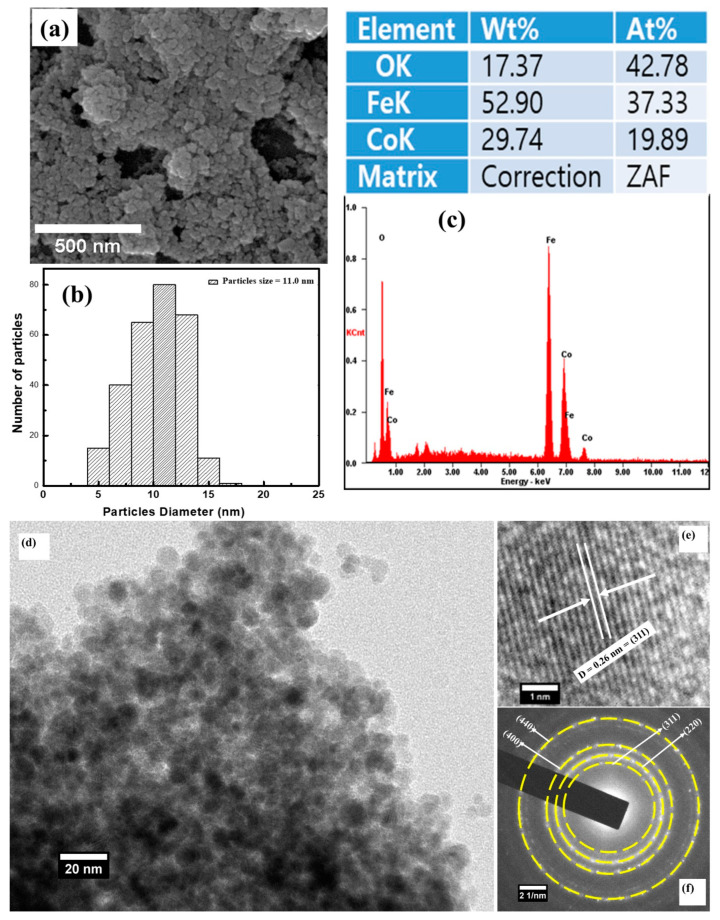
(**a**) Field emission scanning electron microscopy (FE-SEM) micrograph of CeFe_2_O_4_ nanostructures, (**b**) particle size distribution histogram of CeFe_2_O_4_ nanostructures, (**c**) energy-dispersive X-ray spectroscopy (EDS) spectrum of CoFe_2_O_4_ nanostructures and the stoichiometry (%) of constituent elements present in CeFe_2_O_4_ nanostructures. (**d**) Transmission electron microscopy (TEM) micrograph of CeFe_2_O_4_ nanostructures, (**e**) high-resolution transmission electron microscopy (HR-TEM) micrograph of CeFe_2_O_4_ nanostructures, (**f**) selective area electron diffraction (SAED) pattern of CeFe_2_O_4_ nanostructures.

**Figure 3:**
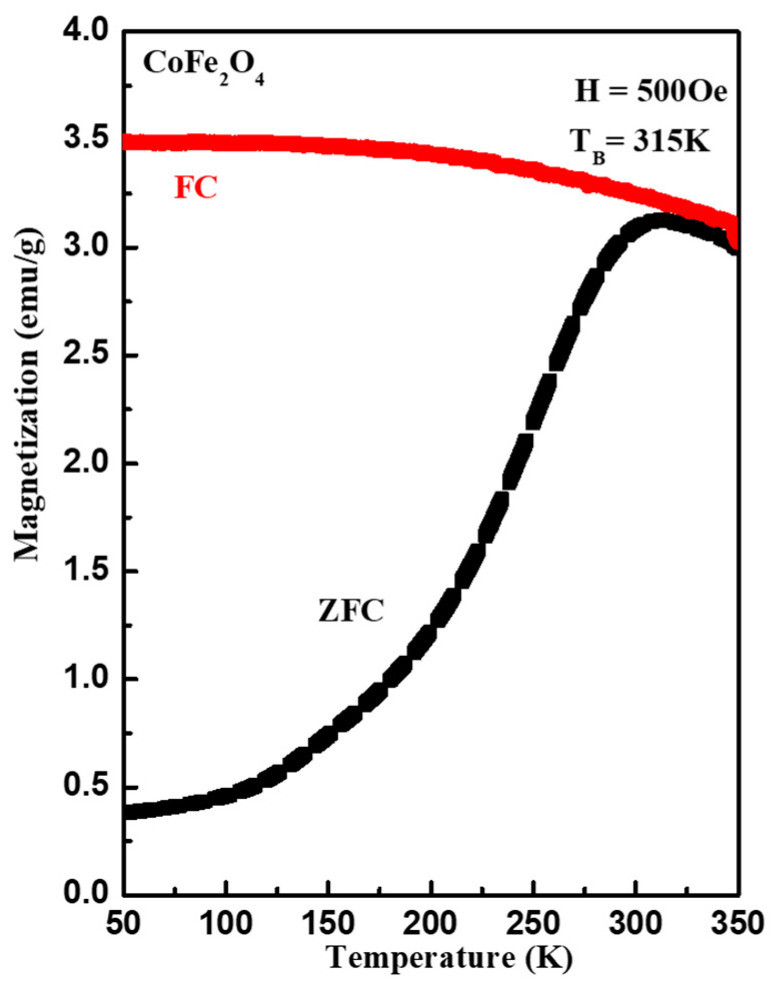
Field-cooled (FC) and zero-field-cooled (ZFC) magnetization versus temperature curves of CeFe_2_O_4_ nanostructures measured in a magnetic field of 500 Oe.

The surface morphology of the CoFe_2_O_4_ nanostructures recorded using field emission electron microscopy (FE-SEM) is shown in Figure 2a. The FE-SEM image shows that CoFe_2_O_4_ exhibited a spherical shape morphology and revealed its nanocrystalline nature. The particle size calculated from the FE-SEM micrograph (see Figure 2b) using Image-J software was found to be 11.0 nm. The EDS spectrum of the CoFe_2_O_4_ nanostructures is displayed in Figure 2c. The ratio of Co:Fe (Figure 3c) observed from the EDX spectrum was ~1:2, indicating that the stoichiometric proportion was maintained. 

Additionally, the morphology of CoFe_2_O_4_ nanostructures was further determined using transmission electron microscopy (TEM), as shown in Figure 2d. The TEM micrograph reveals that CoFe_2_O_4_ had a nanocrystalline behavior and is in good agreement with the FE-SEM and XRD results. In addition, the crystal structure of the CoFe_2_O_4_ nanostructures was again confirmed using high-resolution transmission electron microscopy and the selective area electron diffraction (SAED) pattern. The rings observed in the SAED pattern (see Figure 2f) were successfully recognized and assigned to (220), (311), (400), and (440) orientations. The interplanar distance determined using HR-TEM (see Figure 2e) was observed to be 0.26 nm, which matches with the (311) plane of the FCC crystal structure. Hence, it is possible to say that the HR-TEM, SAED, and XRD results signify that the CoFe_2_O_4_ nanostructures were successfully prepared, without any impurity phase.

Figure 3 demonstrates the magnetization versus temperature (M-T) measurement of the CoFe_2_O_4_ nanostructures. The M-T measurements, performed in zero-field-cooled (ZFC) and field-cooled (FC) measurements mode, followed a procedure in which the sample was cooled from 300 K to 50 K in the absence of (ZFC); thereafter, a static magnetic field of 500 Oe was applied and the magnetization was recorded during the warming-up cycle. Then, the sample was cooled from 300 K to 50 K in the presence of the magnetic field of 500 Oe (FC) and the magnetization was measured in the warming-up cycle. The ZFC–FC measurements indicated that the CoFe_2_O_4_ nanostructures showed ferromagnetic ordering at room temperature, with a blocking temperature (T_B_) of 315 K, which is a transition from a superparamagnetic state to a blocked state. Here, it is worth mentioning that a change in FC and ZFC magnetization below 315 K resulted from the energy barriers of the magnetic anisotropy [30]. The T_B_ of magnetic materials depends on the interparticle magnetic interactions, as well as particle size. With the help of T_B_ and the volume of the single particle, the magnetic anisotropy constant K was calculated using the formula (K=25KBTBV) and observed to be 3.2 × 10^5^ J/m^3^, where $K_{B}$ is Boltzmann’s constant.

**Figure 4:**
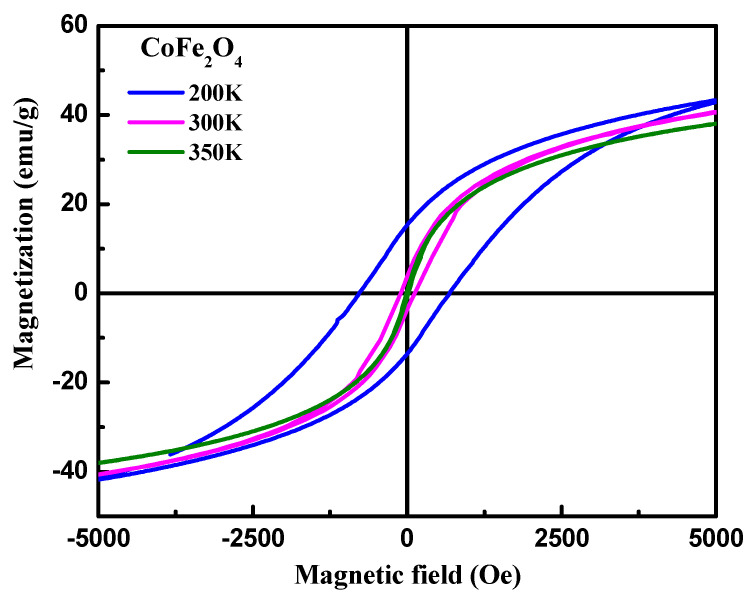
Hysteresis loops of CeFe_2_O_4_ nanostructures at different temperatures.

Figure 4 represents the magnetic hysteresis (M-H) loop of CoFe_2_O_4_ nanostructures measured at different temperatures. It can be observed that the CoFe_2_O_4_ nanostructures demonstrated ferromagnetic behavior at temperatures 200 K and 300 K, whereas the reversible M-H curve at 350 K reflects a superparamagnetic behavior. From Figure 4, it is evident that the area under the hysteresis loop increased with a decrease in the temperature. The values coercive field (H_C_) and remanence magnetization (M_R_) calculated from the hysteresis loop were 755 Oe and 16 emu/g at 200 K, and 91.0 Oe and 3.7 emu/g at 300 K, respectively. It can be observed from Figure 4 that the magnetization was unsaturated until applying 5 T. Therefore, the M_S_ was calculated by extrapolating the M versus H/T curves. The saturation magnetizations (M_S_) calculated using M-H loops were 50.0 emu/g, 48.0 emu/g, and 46.0 emu/g at 200 K, 300 K, and 350 K, respectively. The values of the various magnetic parameters (see Table 1) were found to decrease with an increase in temperature. This can be attributed to the decrease in the exchange interactions between the spin moments, because of the thermal activation energy. 

**Figure 5:**
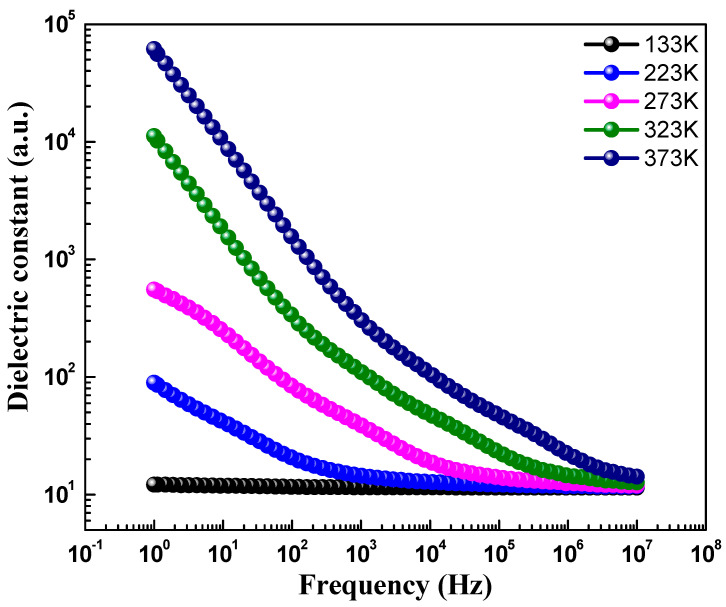
Dielectric constant versus frequency curve of CeFe_2_O_4_ nanostructures at different temperatures.

**Figure 6:**
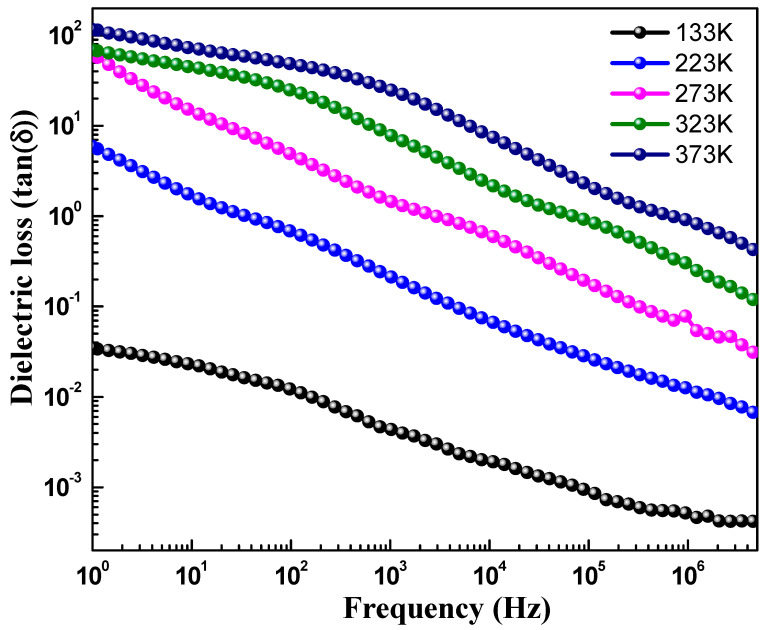
Loss tangent (tanδ) versus frequency curve of CeFe_2_O_4_ nanostructures at different temperatures.

Figure 5 depicts the dielectric constant (ε′) versus the frequency curve, showing the real part of the dielectric constant of CoFe_2_O_4_ nanostructures measured at different temperatures in the frequency range of 1.0 Hz to 10 MHz. The value of the dielectric constant was determined using the formula ε′=Cp×dε0×A, where *d* is the thickness of the pellet, $C_{p}$ is the capacitance of the parallel pellet, A is the cross-sectional area of the painted surface, and $\varepsilon_{0}$ is the space permittivity of free space. The value of ε′, as shown in Table 2, was found to increase with the increase in the temperature. Additionally, the dielectric constant was found to decrease, as observed in Figure 5, with an increase in frequency, which is the usual behavior reported for most polar dielectric materials [31]. However, it was noticed that the decreasing behavior became slower at the higher frequencies and showed an almost frequency-independent behavior. This type of frequency-dependent nature of ε′ can be interpreted using the Maxwell–Wagner two-layer model, which is consistent with Koop’s theory of dielectrics [15]. Koop’s model explains that dielectric materials are made-up of conducting grains, as well as insulating grain boundaries. It is reported that grain boundaries result in ferrites because of the oxidation of superficial reduction of crystallites as a result of the sintering process [32,33,34,35]. When dielectric measurements are performed in the alternating field, the grain boundaries dominate at lower frequencies, whereas high conductive grains are effective at high frequencies. Therefore, the voltage applied to the specimen drops predominantly on both sides of the grain boundaries and causes space charge polarization, since the hopping of electrons inside the material accumulates at the grain boundaries because of high grain boundary resistance. The large value of the ε′ in CoFe_2_O_4_ nanostructures is ascribed to the greater effect grain boundaries have over grains than at lower frequencies, which indicates the major role of space charge polarization. However, the ε′ starts to decrease as the frequency increases further, because of the weak space charge polarization. At high frequencies, electrons lag behind, while following the changes of the applied field exchange, and thus ε′ decreases and become steady beyond a certain frequency limit. Figure 6 highlights dielectric loss (tanδ) versus frequency of CoFe_2_O_4_ nanostructures. The dielectric loss was measured in the frequency range of 1.0 Hz to 10 MHz at different temperatures. The dielectric loss tangent ((tanδ) can be determined using the formula tanδ=ε″ε′, where $\varepsilon^{\prime \prime }$ is the complex dielectric constant. The dielectric loss tangent (tanδ) signifies the loss of energy from the applied field into the material and that is dissipated in the form of heat energy. It was found that the dielectric loss decreased with an increase in frequency of the applied field, indicating a normal dispersion behavior. It can be seen from Figure 6 that tanδ has a low loss value at higher frequencies, which shows its suitability for applications in high frequency devices. The high value of tanδ at low frequency may be because of its high resistivity (owing to grain boundary). Since the electrons’ hopping among Fe^2+^ ⇔ Fe^3+^, as well as hole transfer involving Co^2+^ ⇔ Co^3+^, require more energy, this causes the high value of tanδ. Moreover, in the high frequency region, the hopping of electrons requires little energy, due to the low resistivity (because of grains), and as a result, the loss is small. Furthermore, the value of tanδ is also dependent on other factors, such as the sintering temperature of the material, composition, structural homogeneity, and the Fe^2+^ and Fe^3+^ content in samples. Additionally, it can be noticed that the values ε′ and tanδ increase with temperature and display a semiconducting behavior (See Table 2). This may be due to the fact that, at low temperatures, the charge carriers do not have sufficient energy and because of that cannot orient themselves along the course of the applied field variation and, hence, contribute weakly to the polarization. An increase in temperature causes the charge carries to obtain sufficient thermal energy and they start to follow the change in the externally applied field, and as a result of this, the dielectric constant increases. 

**Figure 7:**
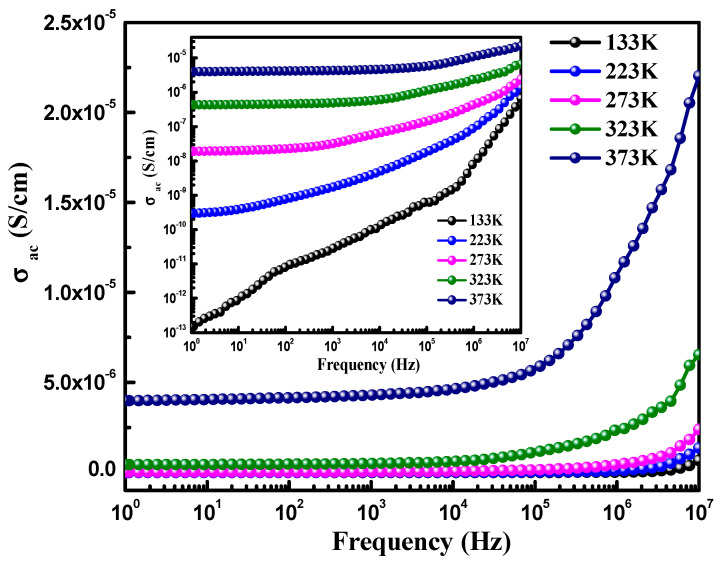
σ_ac_ versus frequency curve of CeFe_2_O_4_ nanostructures at different temperatures. Inset shows log (σ_ac_) versus log (frequency) curve of CeFe_2_O_4_ nanostructures at different temperatures.

Furthermore, the ac-conductivity observed in the dielectric materials can be measured in relation with the frequency using the following equation: σ_ac_ = є’ є_o_ω tanδ, where the symbols have their usual meaning [36,37]. The ac conductivity was observed to increase with an increase in the temperature (see Table 2). The frequency-dependent ac-conductivity curves of CoFe_2_O_4_ are displayed in Figure 7, in the frequency range of 1.0–10.0 MHz. The curves demonstrate that the ac-conductivity substantially follows the frequency, and showing a good correlation between the two. The increase of ac-conductivity with frequency may be associated with the possible reduction in the resistance present at the grain boundaries, which was developed due to the charge accumulation at the opposite sides of the grain boundaries. The short-grain boundaries may have reduced the resistance and enhanced the ac-conductivity with the frequency. 

## 4. Conclusions

We successfully prepared CoFe_2_O_4_ nanostructures using a microwave-supported hydrothermal route. The crystal structure studied using HR-TEM, XRD, and SAED spectra confirmed the single-phase nature, with an FCC structure. Crystallographic parameters, such as the lattice parameters and unit cell volume, calculated using Rietveld refinement of XRD data were 8.4821 Å and 583.88 Å^3^, respectively. The average crystallite size measured using the FE-SEM and XRD data was 11.0 nm and 7.0 nm, respectively. The spherical type morphology of CoFe_2_O_4_ nanostructures was observed from the TEM and FE-SEM micrographs. The ZFC and FC studies of CoFe_2_O_4_ nanostructures indicated ferromagnetic ordering at room temperature, along with a T_B_ of ~315 K. The M-H measurements showed that the CoFe_2_O_4_ nanostructures demonstrated a superparamagnetic behavior above room temperature. It was observed that with an increase in frequency, the dielectric constant and loss tangent were found to decrease. However, the dielectric constant and loss tangent were observed to increase with an increase in temperature.

## Figures and Tables

**Table 1:** Saturation magnetization (Ms), and remanence magnetization (M_R_), coercive field (H_C_) of CeFe_2_O_4_ nanostructures calculated from M-H curve at different temperatures.

	200 K	300 K	350 K
M_S_ (emu/g)	50	48	46
M_R_ (emu/g)	16	3.7	0
H_C_ (Oe)	755	91	0

**Table 2:** Dielectric constant (ε′), loss tangent (tanδ), and ac conductivity (σ_ac_) CoFe_2_O_4_ nanostructures recorded at 1.0 MHz.

CoFe_2_O_4_	133 K	233 K	273 K	323 K	373 K
Dielectric constant (ε′)	11.4	11.8	12.4	14.3	20.8
Loss tangent (tanδ)	4.6 × 10^−4^	1.4 × 10^−3^	5.0 × 10^−2^	0.23	0.82
ac conductivity (σ_ac_)	1.2 × 10^−8^	1.1 × 10^−7^	4.8 × 10^−7^	2.4 × 10^−6^	1.2 × 10^−5^

## Data Availability

Available on request.
